# Patient and public involvement in clinical trials to improve outcomes for adults with multimorbidity in primary care and community settings: A systematic review protocol

**DOI:** 10.1177/26335565261427228

**Published:** 2026-03-26

**Authors:** Elizabeth A. O’Donnell, Zara Khwaja, Susan M. Smith, Carmel P. Geoghegan, Martin Quinn, Laura J. Sahm, Edel Burton, Ann Sinéad Doherty, Emma Wallace

**Affiliations:** 1 Department of General Practice, School of Medicine, 8795 University College Cork, Cork, Ireland; 2 Discipline of Public Health and Primary Care, School of Medicine, 8809 Trinity College Dublin, Dublin, Ireland; 3 PPI Partner, Clinical Pharmacy and General Practice PPI Group, 8795 University College Cork, Cork, Ireland; 4 Pharmaceutical Care Research Group, School of Pharmacy, 8795 University College Cork, Cork, Ireland; 5 School of Public Health, 8795 University College Cork, Cork, Ireland; 6 Pharmacy Department, 155290 Bon Secours Hospital, Cork, Ireland; 7 School of Pharmacy and Biomolecular Sciences, 8863 RCSI University of Medicine and Health Sciences, Dublin, Ireland

**Keywords:** patient and public involvement (PPI), clinical trials, randomised controlled trials (RCTs), multimorbidity, primary care, community settings, PPI reporting, systematic review

## Abstract

**Background:**

Patient and public involvement (PPI) in clinical trials for adults with multimorbidity (multiple long-term conditions) in primary care is essential to ensure research is person-centred. However, PPI is often underreported, limiting understanding of its application and impact. This protocol describes a systematic review examining the uptake, impact and reporting quality of PPI in clinical trials of interventions to improve mental health, clinical or quality-of-life outcomes for adults with multimorbidity in primary care.

**Methods:**

The review will be guided by the Cochrane Handbook and reported according to PRISMA-P guidelines. Eligible studies include completed and ongoing randomised and non-randomised controlled trials. Multimorbidity is defined as the co-existence of two or more long-term conditions. Electronic databases (MEDLINE, CINAHL, Embase, Cochrane) will be searched from 2019 to update Smith et al. (2021) without language restrictions. Trial registries and grey literature will identify protocols and supplementary data. Inclusion criteria - Population: adults with multimorbidity; Interventions: targeted at this population; Comparison: usual care; Outcomes: mental health, clinical or quality-of-life; Setting: primary or community care. Studies will be included irrespective of whether PPI was reported. Data extraction will capture PPI presence, characteristics, activities, training and acknowledgement. A narrative synthesis will describe reported PPI in clinical trials. Two PPI partners will contribute throughout the review. The protocol is registered with PROSPERO (CRD420251090082).

**Discussion/Conclusion:**

This review will enhance understanding of PPI in trials aiming to improve outcomes for adults with multimorbidity in primary and community care, identify gaps in reporting, and inform future trials to support person-centred research.

## Background

Patient and public involvement (PPI) refers to ‘research carried out ‘with’ or ‘by’ members of the public rather than ‘to’, ‘about’ or ‘for’ them.’^1^ As such, it values patients as partners in the research process, contributing at multiple different stages, including priority setting, study design, intervention design, selecting relevant outcomes, recruitment, contributing to data collection, analysis, and dissemination.^2^

Integrating PPI into the design of clinical trials is essential for conducting research that is person-centred and grounded in lived experience.^2^ For instance, the UK 3D (Dimensions of health, Drugs and Depression) trial incorporated a panel of fourteen PPI partners in the design and conduct of a cluster randomised controlled trial (RCT) in general practice, evaluating the efficacy of a patient-centred, GP-led approach to managing multimorbidity that focused on implementing holistic reviews, depression screening, and medication optimisation.^3^ The PPI panel advised on the relevance of the research question, trial outcomes, recruitment strategies, qualitative data collection tools, and dissemination activities. PPI partner input led to improvements in the clarity of patient materials and consent procedures. PPI partners also co-developed a tool to measure treatment burden and shaped data analysis. PPI contributions ensured the intervention addressed patient priorities and enhanced patient-centred care as well as patient satisfaction.^3^ This demonstrates the significant benefits of PPI contributions in ensuring trials examine outcomes that are person-centred, relevant and reflect the lived experience of people with multimorbidity.

However, such detailed and transparent reporting of PPI is uncommon. There is currently limited understanding of how consistently PPI input is operationalised at protocol development and trial stages. For instance, a meta-epidemiological review by Vanneste et al. (2025)^4^ explored PPI reporting in 360 RCTs published since 2015 in four leading medical journals (The New England Journal of Medicine, The Lancet, the Journal of the American Medical Association, and The British Medical Journal), along with 299 corresponding protocols.^4^ The review found that only approximately one fifth of trial articles and corresponding protocols reported PPI. Where PPI was reported it usually involved contributions such as feedback on study design, review of patient-facing materials, or membership of steering committees. However, descriptions of PPI involvement lacked detail with little reporting of impact, despite PPI often being a requirement for research funding.^4^ In relation to multimorbidity trials specifically, limitations in PPI reporting were highlighted in the findings of a 2016 systematic review of multimorbidity trials in primary care.^5^

In response to gaps in PPI reporting, frameworks have been developed to improve the quality and transparency of such reporting. The Guidance for Reporting Involvement of Patients and the Public (GRIPP-2), introduced by Staniszewska et al. (2017),^6^ is regarded as the gold-standard guideline for recording PPI contributions in health research. GRIPP-2 provides checklists that support researchers in reporting whether PPI was undertaken, how it was implemented and what impact it had, thereby enhancing consistency in reporting PPI practice.^6^

Yet, as highlighted in the recent review by Vanneste et al. (2025)^4^ even with GRIPP-2 available, PPI reporting remains limited. This lack of detail in reporting PPI input highlights the limited uptake of reporting guidelines such as GRIPP-2, which were developed to strengthen the quality and consistency of PPI reporting.

Currently, the extent and nature of PPI involvement in clinical trials addressing multimorbidity in primary and community care settings remain unknown. Addressing this knowledge gap is important as people with multimorbidity often experience higher treatment burden related to the management of their long-term health conditions.^7^ Additionally, multimorbidity is more prevalent among older adults and for those living in socio-economically disadvantaged areas and is associated with poorer health outcomes and reduced quality of life.^8^ As such, integrating the perspectives of people with lived experience of multimorbidity in the planning and delivery of RCTs is essential for ensuring that research is impactful in real-world settings.

As demonstrated by the 3D trial, PPI involvement can result in the inclusion of trial outcomes that focus on person-centred care.^3^ Hence, embedding PPI in multimorbidity-focused RCTs has the potential to improve outcomes for individuals with multimorbidity. A further consideration is that PPI input is ethically necessary to promote the principles of justice, fairness, and respect.^9^ One example of this is the LinkMM (link workers for people with multimorbidity) pilot trial which evaluated primary care-based link workers providing social prescribing to improve health outcomes for people with multimorbidity attending general practices in socioeconomically deprived areas in Ireland.^9^ The LinkMM pilot trial embedded a PPI advisory group from the outset. PPI partners co-developed recruitment materials, informed selection methods, and guided trial design.^9^ These actions demonstrated a commitment to PPI partnership and the study was reported according to GRIPP-2 guidelines. PPI partnership in trials may be seen as an ethical imperative, essential for designing trials that are equitable and person-centred.

While the LinkMM trial demonstrates the ethical and practical value of meaningful PPI,^9^ many multimorbidity trials encounter real-world challenges that limit how fully PPI is implemented, particularly in terms of recruitment and retention. For example, participants may be older and living with multiple long-term health conditions which can limit their ability to remain involved in the full life cycle of a trial which takes several years from protocol development, funding to trial conduct and reporting.^10^ There is also a challenge in participants recruitment to find representative voices due to the heterogeneity of people living with multimorbidity. Patient representative and advocacy groups may focus on single conditions and ensuring patients from socioeconomically disadvantaged backgrounds are included is also challenging.^11^ Other potential barriers include limited funding to support PPI activities and/or to train contributors, poorly defined roles for PPI members, and a lack of training for researchers on the value of PPI and strategies for sustaining engagement.^12^ These challenges can increase the risk of tokenism.^13^ Tokenism refers to unequal power dynamics or partnering with PPI collaborators but not valuing input as equally important as other research and clinical collaborators, for example, to secure research funding.^13^ Hence, there is need for a more structured and transparent understanding of PPI in multimorbidity research.^12^

## Aim and objectives

### Aim

This systematic review aims to examine the uptake, impact and quality of reporting of patient and public involvement (PPI) in clinical trials and protocols of trials focused on interventions to improve mental health, clinical or health-related quality of life outcomes for adults with multimorbidity in primary care and community settings.

### Objectives

The objectives of this review are to:

**1. Identify** the extent, uptake, impact, quality and reporting of PPI contribution in both clinical trials and trial protocols.
**2. Investigate** the nature of PPI partner engagement by identifying the stages of the research process in which PPI contributions are incorporated (e.g. governance, setting research priorities, formulating research questions, study design, intervention design, selection of outcomes, recruitment, data collection, analysis, or dissemination). Also, the specific contributions made (e.g. co-developing materials or lay translation of results).
**3. Assess** planned PPI activities reported in trial protocols against actual PPI contribution reported in their corresponding published trials.
**4. Inform** planning by synthesising current practice and identifying opportunities to improve PPI contribution in future multimorbidity trials in primary care and community settings.

## Methods

### Study design

This protocol is reported according to Preferred Reporting Items for Systematic Review and Meta-Analysis Protocols (PRISMA-P) for systematic review protocols guidelines (Moher et al. 2015)^14^ (Supplemental file 1). The conduct of the review will be guided by the Cochrane Handbook.^15^

This review is registered with PROSPERO (CRD420251090082).

Ethical approval is not required for systematic reviews.

### Definitions

Multimorbidity, also referred to as multiple long-term conditions, is defined as the co-existence of two or more long-term health conditions in an individual.^16^

Chronic or long-term health condition is defined as conditions that last a year or more and require ongoing medical attention and/or limit activities of daily living (ADL).^17^

Primary care refers to the first point of contact within the healthcare system, provided by general practitioners (GPs), nurses, and other healthcare professionals in community-based practices or health centres.^18^

PPI is defined as the active contribution of patients, carers or public to the design, implementation or conduct of the RCT, in distinct contrast to being enrolled as a participant in an RCT.^19^

### Inclusion criteria

**Completed and ongoing** clinical trials including RCTs, non-RCTs (e.g. controlled clinical trials or quasi-experimental designs), will be included if they meet the following criteria:

• **Population**: Adults ≥18 years with multimorbidity (except for studies focused on a single index condition with defined comorbidities e.g. heart failure with depression). Studies explicitly describing participants as having multimorbidity will be included, even if operational definitions differ. Explicit reporting of ongoing medical attention or ADL limitations is not required (unclear cases will be assessed by two reviewers to reach consensus).
• **Interventions:** Consistent with Smith et al. (2021),^20^ interventions delivered in primary care or community settings targeting adults with multimorbidity through improved care or management approaches will be included (with the exception of professional education delivered solely to clinicians with no direct patient care). Trials with components in secondary or residential care (e.g., discharge planning) are included only if primary aims and outcomes relate to primary or community care.
• **Comparators:** Placebo, usual care or active comparator.
• **Outcomes**: Acceptability, feasibility, clinical, wellbeing, quality of life, mental health or economic outcomes.
• **Study Design:** Completed and ongoing RCTs and non-RCTs trials (e.g. controlled clinical trials or quasi-experimental designs).
• **Publication Type**: Peer-reviewed trials and protocols; trial protocols reported in registries; published quantitative, qualitative and/or mixed-methods trial process evaluations linked to included trials.
• **Date:** This review will update the searches conducted for the systematic review by Smith et al. (2021),^20^ which included studies measuring efficacy or effectiveness of interventions aimed at improving outcomes for adults with multimorbidity in primary care and community settings. The previous review included trials published up to September 2019. Professor Susan Smith, who was the lead author of the 2021 review, is also a co-author of the current review. This review focuses on characterising patient and public involvement (PPI) in clinical trials evaluating interventions to improve mental health, clinical or health-related quality of life outcomes for adults with multimorbidity in primary care and community settings. Accordingly, it will include all new trials conducted over the past six years (since October 2019) in addition to the 16 RCTs included in the review by Smith et al. (2021)^20^
• **Language:** No restrictions
• **PPI Consideration:** Studies will be included irrespective of whether PPI was reported.

### Search strategy

A comprehensive search will be conducted to update a previous systematic review by Smith et al. (2021), which included clinical trials up to September 2019.^20^ This review will include new clinical trials published since 2019, alongside the 16 RCTs identified in Smith et al. (2021).^20^

Four electronic bibliographic databases will be searched

• MEDLINE (via Ovid),
• Embase (via Ovid),
• CINAHL (via EBSCOhost),
• Cochrane Central Register of Controlled Trials (CENTRAL).

In addition, clinical trial registries, ClinicalTrials.gov and the WHO International Clinical Trials Registry Platform (ICTRP) will be searched to identify trial registrations, protocols, and associated publications linked to included studies.

### Grey literature searching will adopt a targeted approach

• Funding repositories and funder websites will be searched for protocols, process evaluations, and reports associated with included trials.
• Forward citation searching and screening of reference lists of included studies will be undertaken to identify additional trials, protocols, and process evaluations.

As this review is an update of the systematic review by Smith et al. (2021)^20^ the original search strategy from that review will be replicated. An example of the key search terms is provided in Table 1 based on the population, intervention, context/setting and study design (PICOS) Framework.

**Table 1.** Outline of search keywords/key terms based on PICOS Framework.

PICOS framework	Search words/terms
**Population:** Adults with multimorbidity	multimorbid*, comorbid*, chronic disease
**Context/setting:** Primary care and community settings	primary health care, general practice, community health services, gp*, family practice, ambulatory
**Interventions:** Any intervention targeting adults with multimorbidity aimed at improving mental health, clinical or health-related quality of life outcomes.	intervention*, program*, care management, self-management, education, multidisciplinary care
**Study design:** Clinical trials and protocols	randomised controlled trial, controlled clinical trial, trial, RCT, random*, multicentre study, trial protocol

The full database search strategies for MEDLINE, Embase, CINAHL and Cochrane CENTRAL are provided in Supplemental File 2. Zotero will be used for reference management.

### Screening and identification of relevant studies

In the initial phase, two reviewers will independently screen titles and abstracts of retrieved studies for eligibility based on the predefined inclusion and exclusion criteria. Rayyan software, a web-based tool, will be used to facilitate the screening process. Articles deemed eligible after this initial screening phase will proceed to full-text review where two reviewers will independently read the full articles to assess eligibility. At both screening stages, any disagreements between reviewers regarding eligibility will be resolved through discussion to reach consensus. If consensus cannot be achieved, an adjudicator (EW) will act as arbitrator if needed. A PRISMA flow diagram will illustrate the number of studies identified, screened, included, and excluded, with reasons for full-text exclusions.^21^

### Data extraction

Data will be extracted from included studies and study protocols using a structured pilot-tested Excel data extraction form to ensure consistency and comprehensiveness. The data extraction form was pilot tested on three studies and iteratively refined to optimise data extraction and presentation. Data from the pilot phase will be included in the final analysis. The data extraction form will collect the following information from full-text articles.

### Study information

• Article title, authors, journal, year of publication, country and setting of care.

### Clinical trial population, intervention and outcomes

• Definition of multimorbidity used and conditions noting alignment with this protocol’s definition.
• Definition of chronic/long-term conditions noting alignment with this protocol’s definition.

### Intervention type and development

• Intervention description using the TIDieR checklist^22^ including the main focus of the intervention:
  oCare coordination plus additional patient-focused support
  oSupport for self-management
  oMedicines management
• Whether the intervention was designed or adapted for adults with multimorbidity (as opposed to a single-condition population).

### Trial participant characteristics

• Participant characteristics including number of long-term conditions, which conditions commonly occurred together, and severity of multimorbidity.

### Primary and secondary outcomes

#### PPI involvement

• Reported PPI engagement and contributions.
• Characteristics of PPI contributors e.g. number, gender, age, role (e.g. patients with multimorbidity, carers or members of the public).
• Whether PPI contributors were individually recruited or part of an established advisory group, if reported.
• PPI activities/processes undertaken and at which stage of research (e.g. protocol development, study feasibility, grant application, formulation of research questions, study design, intervention design, selecting outcome measures, ethical approval, review of study materials, recruitment, data collection, data analysis, interpretation, writing results or publications, reviewing results or publications, co-producing lay-summaries, conference presentations or other dissemination activities).
• Training of PPI contributors
• Recognition of PPI contributions (e.g. reimbursement, acknowledgement, gifts, awards, authorship).
• Reported impact of PPI on study design, implementation, or outcomes.

The authors of included clinical trials will be contacted for clarification on reported PPI, if required. One researcher (EOD) will complete the data extraction. A second reviewer will check all the data extracted for quality assurance purposes (e.g. accuracy or any potential omissions). Completed data extraction forms will be included in final systematic review appendices.

### Quality appraisal of PPI reporting

The GRIPP2-SF (Short Form) checklist (Staniszewska et al. 2017)^6^ (available from the BMJ DOI: 10.1136/bmj.j3453) will be used to appraise the reporting quality of PPI in trial protocols and published trials where PPI is not the primary focus of the study. The GRIPP2-SF (Short Form) checklist evaluates the comprehensiveness and quality of PPI reporting rather than the efficacy of the interventions themselves.^6^ The focus of quality appraisal is on PPI reporting, not the methodological quality of the trial. Two reviewers will independently assess reporting of PPI in trials, trial protocols and process evaluations using the GRIPP2-SF form. Disagreements will be discussed to achieve consensus with arbitration to a third reviewer (EW), if required.

### Data synthesis

A meta-analysis is not appropriate for this review which explores PPI involvement in multimorbidity clinical trials. Therefore, a narrative synthesis will be undertaken to describe reported patient involvement in clinical trials and their protocols. A narrative synthesis is discursive in nature and seeks to summarise the current state of knowledge in relation to a particular domain by considering a wide variety of sources and reaching conclusions through reason or argument.^23^

This synthesis will collate the nature, extent, impact, and quality of PPI reporting in multimorbidity trials and protocols. As part of this process, we will describe how included studies define multimorbidity and consider the consistency of these definitions with this review’s eligibility criteria. This will allow meaningful interpretation of variation in PPI reporting across studies and enhance understanding of how PPI is planned, implemented, and reported in multimorbidity research. One researcher (EOD) will lead the synthesis. Any uncertainties during synthesis will be resolved through consultation with the wider research and clinical team members to ensure reliability and trustworthiness.

### Patient and public involvement

This study will be supported by two PPI contributors (MQ and CG) with lived experience of multimorbidity. They will provide feedback throughout all stages of this systematic review. Specifically, the PPI contributors have reviewed the study protocol to ensure the relevance of the research aim and objectives. They have also assessed the clarity and readability of the protocol. In addition, the PPI contributors will review and interpret the systematic review findings to help to put the results in context. PPI will also input into the drafting and publication of the review to enhance overall clarity. The PPI collaborators will be actively involved in co-producing a lay-summary and other dissemination activities to ensure that review findings are relevant. We will report on these PPI activities in the review itself using the GRIPP-2 SF checklist. PPI partners (MQ and CG) contributions will be recognised through co-authorship.

## Discussion

This systematic review protocol outlines the planned approach to investigate the nature, extent, impact and quality of reporting of PPI in clinical trials, protocols (and process evaluations) focused on improving outcomes (e.g., mental health, clinical or quality of life) for adults with multimorbidity in primary care and community settings, through improved care or management approaches. This review should improve current knowledge and understanding of how PPI is incorporated into trials in this area and highlight the degree and consistency of reporting between protocols and published trials.

Strengths of this review protocol include a robust methodology reported via the PRISMA-P for systematic review protocol guidelines. Two reviewers will be involved in screening, and quality appraisal. A second reviewer will also check the accuracy of all the data extracted. Any uncertainties during synthesis will be resolved through consultation with the wider research and clinical team members to ensure reliability and trustworthiness. Additionally, GRIPP2-SF, used in this review, is a validated tool for assessing the quality of PPI reporting. The narrative synthesis approach is appropriate given the likely heterogeneity of reporting styles and PPI activities. A further strength is the inclusion of both protocols and corresponding published trials, which allows comparison of planned versus actual PPI involvement. Finally, PPI contributors with lived experience of multimorbidity were actively involved in reviewing and advising the design of the study protocol and will review the findings reinforcing its relevance and impact.

However, the protocol also has some limitations. The review will depend on the quality of PPI reporting in the included trials and their protocols. This may, potentially, result in underestimating the extent of PPI engagement, where reporting is suboptimal. To address this, trial authors will be contacted where PPI information is unclear in the publication reducing the risk of underestimating PPI activity. Additionally, while GRIPP2 evaluates the quality of PPI reporting, it does not assess the quality of trials included in the review.

## Conclusion

The systematic review will address a significant knowledge gap by improving understanding of how PPI is reported in clinical trials focused on improving outcomes for patients with multimorbidity in primary care and community settings. The findings will inform future planning for PPI engagement in trials in this area, ensuring research is meaningful, person-centred, and impactful.

## Supplemental material

Supplemental material - Patient and public involvement in clinical trials to improve outcomes for adults with multimorbidity in primary care and community settings: A systematic review protocol Supplemental material for Patient and public involvement in clinical trials to improve outcomes for adults with multimorbidity in primary care and community settings: A systematic review protocol by Elizabeth A. O’Donnell, PhD, Zara Khwaja, Susan M. Smith, MD, Carmel P. Geoghegan, Martin Quinn, Laura J. Sahm, PhD, Edel Burton, PhD, Ann Sinéad Doherty, PhD, Emma Wallace, PhD in Journal of Multimorbidity and Comorbidity.

Supplemental material - Patient and public involvement in clinical trials to improve outcomes for adults with multimorbidity in primary care and community settings: A systematic review protocol Supplemental material for Patient and public involvement in clinical trials to improve outcomes for adults with multimorbidity in primary care and community settings: A systematic review protocol by Elizabeth A. O’Donnell, PhD, Zara Khwaja, Susan M. Smith, MD, Carmel P. Geoghegan, Martin Quinn, Laura J. Sahm, PhD, Edel Burton, PhD, Ann Sinéad Doherty, PhD, Emma Wallace, PhD in Journal of Multimorbidity and Comorbidity.

## Data Availability

All materials underlying this systematic review protocol, including the complete PRISMA-P checklist and the full search strategies for each database, are available in the supplementary files associated with this protocol. No primary data will be collected or analysed in this review.*
