# Supplementary material for: Patient and public involvement in clinical trials to improve outcomes for adults with multimorbidity in primary care and community settings: A systematic review protocol
Source: J Multimorb Comorb. 2026 Mar 26;16:26335565261427228. doi: 10.1177/26335565261427228 (PMC13022320; doi:10.1177/26335565261427228)
Supplement: Supplemental material - Patient and public involvement in clinical trials to improve outcomes for adults with multimorbidity in primary care and community settings: A systematic review protocol [file sj-pdf-1-cob-10.1177_26335565261427228.pdf]

Supplementary file 1 - PRISMA-P 2015 Checklist for Systematic Review Protocols

PRISMA-P 2015 Checklist for Systematic Review Protocols

Section/Topic	Item No.	Checklist Item	Reported on Page #
ADMINISTRATIVE INFORMATION			
Title	1a	Identify the report as a protocol of a systematic review.	P. 1
	1b	If the protocol is for an update of a previous systematic review, identify as such.	N/A
Registration	2	If registered, provide the name of the registry (e.g., PROSPERO) and registration number.	P. 1, P. 3
Authors	3a	Provide name, institutional affiliation, and e-mail address of all protocol authors; provide physical mailing address of corresponding author.	P1
	3b	Describe contributions of protocol authors and identify the guarantor of the review.	P8
Amendments	4	If the protocol represents an amendment of a previously completed or published protocol, identify as such and list changes; otherwise, state plan for updates.	N/A
Support	5a	Indicate sources of financial or other support for the review.	P. 8
	5b	Provide name for the funder/sponsor.	P. 8

Supplementary file 1 - PRISMA-P 2015 Checklist for Systematic Review Protocols

	5c	Describe roles of funder(s), sponsor(s), and/or institution(s), if any, in developing the protocol.	P. 8
INTRODUCTION			
Rationale	6	Describe the rationale for the review in the context of what is already known.	P. 2, P. 3
Objectives	7	Provide an explicit statement of the question(s) the review will address with reference to participants, interventions, comparators, and outcomes (PICO).	P. 3
METHODS			
Eligibility Criteria	8	Specify the study characteristics (e.g., PICO, study design, setting, time frame) and report characteristics (e.g., years considered, language, publication status).	P. 4
Information Sources	9	Describe all intended information sources (e.g., databases, trial registers, contact with study authors) with planned dates of coverage.	P. 4, P.5
Search Strategy	10	Present draft of search strategy to be used for at least one electronic database, including planned limits, so it could be repeated.	P.4, P.5, Table 1 (P. 5) Full database search strategies in Supplementary File 2
Study Records	11a	Describe mechanisms to manage records and data throughout the review.	P. 5, P.8
	11b	State the process that will be used for	P.5

		selecting studies (e.g., two independent reviewers) through each phase of the review.	
	11c	Describe planned method of extracting data from reports (e.g., piloting forms, done independently), and any processes for obtaining and confirming data.	P. 5, P. 6
Data Items	12	List and define all variables for which data will be sought (e.g., PICO items, funding sources) and any assumptions and simplifications made.	P. 5, P.6
Outcomes and Prioritization	13	List and define all outcomes for which data will be sought, including prioritization of main and additional outcomes, with rationale.	P.6
Risk of Bias in Individual Studies	14	Describe anticipated methods for assessing risk of bias of individual studies, including whether this will be at outcome or study level, or both.	P. 6
Data Synthesis	15a	Describe criteria under which study data will be quantitatively synthesized.	P. 6
	15b	If data are appropriate for quantitative synthesis, describe planned summary measures, methods of handling data, and methods of	N/A

Supplementary file 1 - PRISMA-P 2015 Checklist for Systematic Review Protocols

		combining data from studies.	
	15c	Describe any proposed additional analyses (e.g., sensitivity or subgroup analyses, meta-regression).	N/A
	15d	If quantitative synthesis is not appropriate, describe the type of summary planned.	P. 6
Meta-bias(es)	16	Specify any planned assessment of meta-bias(es) (e.g., publication bias, selective reporting).	N/A
Confidence in Cumulative Evidence	17	Describe how the strength of the body of evidence will be assessed (e.g., GRADE).	N/A
